# Role of Macrophage Migration Inhibitory Factor in the Proliferation of Smooth Muscle Cell in Pulmonary Hypertension

**DOI:** 10.1155/2012/840737

**Published:** 2012-01-18

**Authors:** Bo Zhang, Min Shen, Min Xu, Li-Li Liu, Ying Luo, Dun-Quan Xu, Yan-Xia Wang, Man-Ling Liu, Yi Liu, Hai-Ying Dong, Peng-Tao Zhao, Zhi-Chao Li

**Affiliations:** ^1^Department of Pathology, Xijing Hospital, Fourth Military Medical University, Changle West Road 169, Xi'an 710032, China; ^2^Department of Pathology and Pathophysiology, Fourth Military Medical University, Xi'an 710032, China; ^3^Department of Cardiology, Xijing Hospital, Fourth Military Medical University, Xi'an 710032, China; ^4^Department of Oncology, Tangdu Hospital, Fourth Military Medical University, Xi'an 710032, China

## Abstract

Pulmonary hypertension (PH) contributes to the mortality of patients with lung and heart diseases. However, the underlying mechanism has not been completely elucidated. Accumulating evidence suggests that inflammatory response may be involved in the pathogenesis of PH. Macrophage migration inhibitory factor (MIF) is a critical upstream inflammatory mediator which promotes a broad range of pathophysiological processes. The aim of the study was to investigate the role of MIF in the pulmonary vascular remodeling of hypoxia-induced PH. We found that MIF mRNA and protein expression was increased in the lung tissues from hypoxic pulmonary hypertensive rats. Intensive immunoreactivity for MIF was observed in smooth muscle cells of large pulmonary arteries (PAs), endothelial cells of small PAs, and inflammatory cells of hypoxic lungs. MIF participated in the hypoxia-induced PASMCs proliferation, and it could directly stimulate proliferation of these cells. MIF-induced enhanced growth of PASMCs was attenuated by MEK and JNK inhibitor. Besides, MIF antagonist ISO-1 suppressed the ERK1/2 and JNK phosphorylation induced by MIF. In conclusion, the current finding suggested that MIF may act on the proliferation of PASMCs through the activation of the ERK1/2 and JNK pathways, which contributes to hypoxic pulmonary hypertension.

## 1. Introduction

Chronic pulmonary hypertension (PH) is a disease characterized by a sustained pulmonary arterial pressure with increases in pulmonary vascular resistance [1]. The pathogenesis of PH has been ascribed to two mechanisms, the initial event of vasoconstriction followed by remodeling of small- and medium-sized pulmonary arteries which is a hallmark of severe and advanced pulmonary hypertension. The main pathological change related to vascular remodeling is an abnormal pulmonary artery smooth muscle cells (PASMCs) hypertrophy and proliferation resulting in obstruction of small pulmonary arteries [2, 3]. Recently, more attention has been given to the facts that pulmonary inflammation could contribute to hypoxic vasoconstriction and remodeling. It has been reported that inflammatory cell infiltrates in the areas of plexiform lesions in human severe chronic pulmonary arterial hypertension [4]. Circulating inflammatory and/or progenitor cells contribute to hypoxia-induced pulmonary vascular remodeling [5, 6]. In addition, accumulating evidence confirms that chronic hypoxia results in the increased expression of lung inflammatory cytokines and chemokines, including interleukin-1 (IL-1), interleukin-6 (IL-6), tumor-necrosis-factor-*α* (TNF-*α*), and Fractalkine, which may potentiate the development of PH [7–10]. For instance, IL-6 promotes the development and progression of pulmonary vascular remodeling and PH through proproliferative antiapoptotic mechanisms [8]. Fractalkine may contribute to PASMCs proliferation in PH [10]. Therefore, there is a growing interest in inflammatory mediators in the pulmonary hypertensive process.

Macrophage migration inhibitory factor (MIF) was originally identified as a T-cell-derived cytokine that inhibits the random migration of macrophages [11]. Currently, MIF is proved to be an important proinflammatory cytokine, secreted by most of the cells including T cells, macrophages/monocytes, endothelial cells, and smooth muscle cells and induce the production of a large number of inflammatory mediators, such as TNF-*α*, IL-1*β*, IL-6, and IL-8 [12, 13]. Thus, many studies revealed MIF to be involved in the pathogenesis of inflammatory diseases, such as atherosclerosis, rheumatoid arthritis, sepsis, asthma, and acute respiratory distress syndrome [14–18]. Besides, MIF may act beyond inflammatory cytokine, as suggested by its proliferative effect on vascular smooth muscle cells demonstrated in atherosclerosis [19]. The evidence provides an idea that MIF might play a role in the vascular disease. However, there is no report that MIF participates in the pulmonary vascular remodeling during exposure to chronic hypoxia.

In the present study, we hypothesized that MIF may have a role in pulmonary vascular remodeling. For that purpose, the expression of MIF was examined in the lungs of hypoxic pulmonary hypertensive (HPH) rats. Then we investigated the effect of MIF on hypoxia-induced PASMCs proliferation and the underlying mechanism.

## 2. Material and Methods

### 2.1. Animals, Drugs, and Chemicals

Male Sprague-Dawley rats (body weight 200–250 g) were used. All of the experimental procedures were approved by the Animal Use and Care Committee for Research and Education of the Fourth Military Medical University. Recombinant mouse MIF was purchased from R&D Systems (Minneapolis, MN, USA). ISO-1 was obtained from Calbiochem (San Diego, CA, USA). MTT, PD 98059, SB 203580, and SP 600125 were from Sigma (St. Louis, MO, USA). MIF monoclonal antibody against rats MIF was gifted from Dr. Chuan-Min Hu, the Third Military Medical University, Chong Qing, China. Antibodies to ERK1/2 and phospho-ERK1/2, JNK and phospho-JNK were from Cell Signaling (Beverly, MA, USA). The inhibitors were dissolved in dimethyl sulfoxide (DMSO). The final amount of DMSO in the bath solution was less than 0.1%.

### 2.2. Treatment of Rat

According to the previous reports, rats were housed intermittently in a chamber containing 10% oxygen, for exposure to continuous hypobaric hypoxia [20]. The intermittent regime consisted of 10 hours in the hypoxic chamber followed by 14 hours in room air (21% oxygen). Rats were exposed to these conditions for a total of 28 days. The normoxic control rats were housed continuously in room air. At the end of hypoxia exposure, measurement of the right ventricle systolic pressure (RVSP) and the ratios of right ventricle/[left ventricle + septum] (RV/[LV+S]) weight were determined. Increases in RVSP and RV/[LV+S] were taken as indicators of pulmonary hypertension.

### 2.3. Cell Culture and Treatment

Rats PASMCs were cultured from explants as previously described [20]. Briefly, as soon as median sternotomy was performed, lungs were removed with hearts in fresh PBS. Under a dissecting microscope, the 2nd-3rd-division (external diameter <300 *μ*m) pulmonary arteries were isolated carefully. After the adventitial layers together with the surrounding tissue and endothelium were removed, the pulmonary arteries were dissected into small pieces and cultured in DMEM supplemented with 100 U/mL penicillin, 0.1 mg/mL streptomycin, 2 mM L-glutamine, and 10% FBS and grown in humidified incubators at 37°C in 95% O_2 _and 5% CO_2_. Cells were used for experiments between passages 3 and 6. In the hypoxic groups, PASMCs were transferred into a hypoxic chamber containing 2% O_2_, 5% CO_2_, and 93% N_2_ for 24 hours. Before exposure to hypoxia or treatment with different agents, cells were undergone serum starvation for 36 hours. Cells were exposed to hypoxia or treated with different agents in 1% FBS DMEM.

### 2.4. Immunohistochemistry

Right lung sagittal sections were placed in 4% paraformaldehyde and processed for paraffin embedding. Sections (5 *μ*m) were cut and mounted on the glass slides. Endogenous peroxidase activity was quenched with 3% peroxide for 10 minutes. The sections were incubated overnight at 4°C with anti-MIF antibody. Slides were washed and incubated with the corresponding secondary antibodies conjugated with alkaline phosphatase.

### 2.5. RT-PCR

Lungs were homogenized, and total RNA was extracted from the lung tissues by using the RNeasy Total RNA Isolation Kit (Qiagen, Valencia, CA). The primers for the rat MIF gene were sense, 5′-TCTCCGCCACCATGCCTATG-3′, and antisense, 5′-GGGTCGCTCGTGCCACTAAA-3′, and for the housekeeping gene *β*-actin were sense, 5′-ATCATGTTTGAGACCTTCAACA-3′, and antisense, 5′-CATCTCTTGCTCGAAGTCCA-3′. PCR reaction was carried out under the following conditions: 30 cycles of denaturation at 94°C for 30 s, annealing at 56°C for 30 s, and extension at 72°C for 30 s. A final extension was performed at 72°C for 1 min. PCR products were separated by 1% agarose gel electrophoresis.

### 2.6. Western Blot Analysis

Total lysates were obtained from harvested lung tissues and cultured PASMCs. Lung homogenates were prepared in RIPA lysis buffer, containing 50 mM Tris (pH 7.4), 150 mM NaCl, 1% Triton X-100, 1% sodium deoxycholate, 0.1% SDS, 2 mM NaF, 5 mM EDTA (pH 8.0), and 1 mM sodium orthovanadate (Beyotime Inc, Jiangsu, China). The protease inhibitor of phenylmethylsulfonyl fluoride (PMSF, 1 mM) was added to the RIPA buffer in advance. Equivalent amounts of protein (30 *μ*g) from each sample were separated on 12% SDS-polyacrylamide gels and then transferred onto 0.22 *μ*m nitrocellulose filter membranes (Millipore, Bedford, USA). The membrane was blocked and incubated with primary antibodies for MIF, or phosphospecific ERK1/2, ERK1/2, phospho-JNK, and JNK. The levels of proteins and phosphoproteins were detected with enhanced chemiluminescent substrate (Pierce, Rockford, IL, USA).

### 2.7. Cell Proliferation Assay

Cells were seeded into 96-well plates and then incubated in medium containing 0.1% DMEM. MIF (50, 100, 200 ng/mL) or PD 98059 (20 *μ*M), SB 203580 (20 *μ*M) and SP 600125 (20 *μ*M) and ISO-1 (10, 50, 100 *μ*M) were added, respectively. After being cultured for 24 hours under normoxic condition or hypoxia exposure, solution MTT was added into each well with a 5 mg/mL concentration. Cells were cultured for another 4 hours, and then dimethyl sulfoxide (DMSO) was added in. After vibrating for 10 minutes, the optical density values were detected at 490 nm wavelength by using a spectrophotometer (Bio-Tek Power Wave XS, USA). Additionally, cell proliferation was evaluated by direct cell counting. PASMCs were seeded in 12-well plates and cultured. Cells were stimulated with MIF or inhibitors as described above, respectively, and after 24 hours they were harvested by mild trypsinization and counted with a hemocytometer.

### 2.8. Statistical Analysis

All values were expressed as mean ± SD. Statistical analysis was processed by using one-way ANOVA, followed by LSD test for post hoc multiple comparisons (SPSS for Windows version 16.0, Chicago, USA). Differences were considered to be significant when *P* < 0.05. 

## 3. Results

### 3.1. Haemodynamics and Right Ventricular Hypertrophy

Rats exposed to hypoxia for 28 days developed pulmonary hypertension (Table 1), as demonstrated by an increase in RVSP (48 ± 2.8 mmHg in hypoxic rats versus 23 ± 0.5 mmHg in control rats) (*n* = 8, *P* < 0.05) and the ratios of RV/(LV+S) weight (0.41 ± 0.05 in hypoxic rats versus 0.26 ± 0.02 in control rats) (*n* = 10, *P* < 0.05).

### 3.2. MIF Expression in Rat Lungs

**Figure 1:**
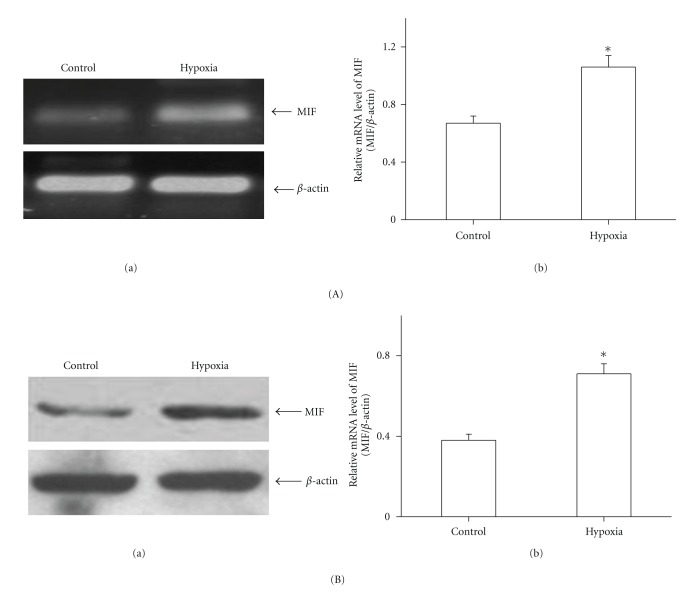
MIF expression in rat lung homogenates. (A) MIF expression was measured by RT-PCR before and after hypoxia exposure. There was a marked upregulation of MIF in the lungs from hypoxic rat (a). Bar graph showed MIF mRNA levels obtained from quantitative densitometry analysis (b). (B) Western blot analysis demonstrated that MIF protein was increased in rat lung homogenates after hypoxia exposure (a). Bar graph showed MIF protein levels obtained from quantitative densitometry analysis (b). *n* = 5. *P < 0.05 versus control.

**Figure 2:**
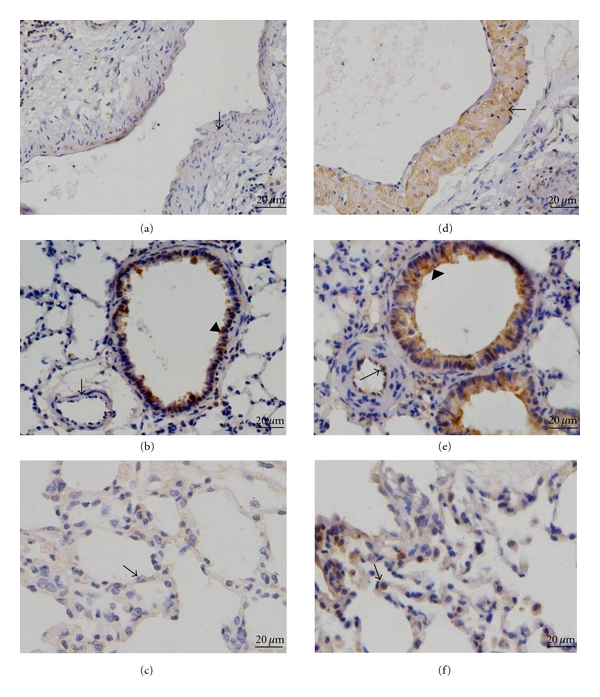
Immunohistochemical analysis of MIF in rat lungs. Immunostaining with an antibody to MIF of normoxic lungs ((a)–(c)) compared with lungs from hypoxic rats ((d)–(f)). The results showed no positive immunoreactivity for MIF in the pulmonary vasculature in control rats (arrow in (a) and (b)), but MIF stained smooth muscle cells of large pulmonary arteries (arrow in (d)), endothelial cells of small pulmonary arteries (arrow in (e)), and inflammatory cells around the alveoli (arrow in (f)) strongly in hypoxic lung sections. Arrows in (c) reveal normal alveoli. Arrowheads in (b) and (e) depict intense staining of bronchial epithelial cells.

Lung homogenates from hypoxic rats showed increases in both MIF mRNA (Figure 1(A)) and protein (Figure 1(B)) compared with control rats. As the immunohistochemistry results show, there was MIF staining in bronchial epithelial cells (Figure 2(b)), but no positive immunoreactivity for MIF in the pulmonary vasculature in control rats (Figures 2(a) and 2(b)), while intense MIF staining of smooth muscle cells of large pulmonary arteries (diameter >100 *μ*m) (Figure 2(d)), endothelial cells of smaller PA (diameter <100 *μ*m), and bronchial epithelial cells (Figure 2(e)), as well as the inflammatory cells around the alveoli (Figure 2(f)), was observed in hypoxic lungs.

### 3.3. MIF-Induced Proliferation of Rat PASMCs

**Figure 3:**
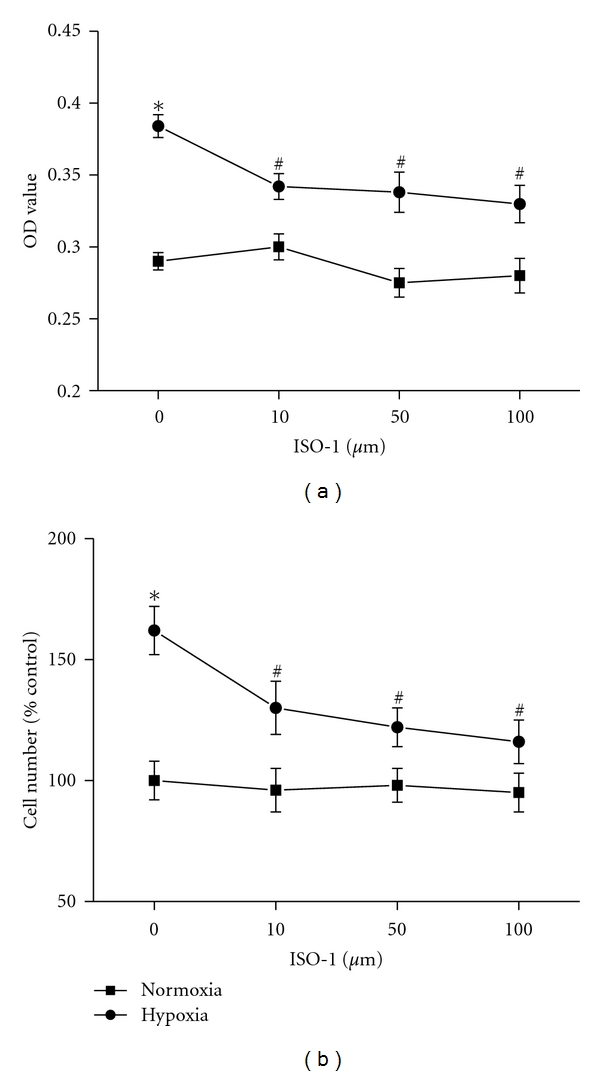
Effects of MIF inhibition on rat PASMCs proliferation under hypoxia exposure. PASMCs proliferation was measured by MTT (a) and direct cell counting (b). Hypoxia-induced PASMCs proliferation was inhibited by different concentrations of MIF antagonist ISO-1 (10, 50, 100 *μ*M). *n* = 6. *P < 0.05 versus normoxic control; ^#^ *P* < 0.05 versus vehicle-treated hypoxia group.

**Figure 4:**
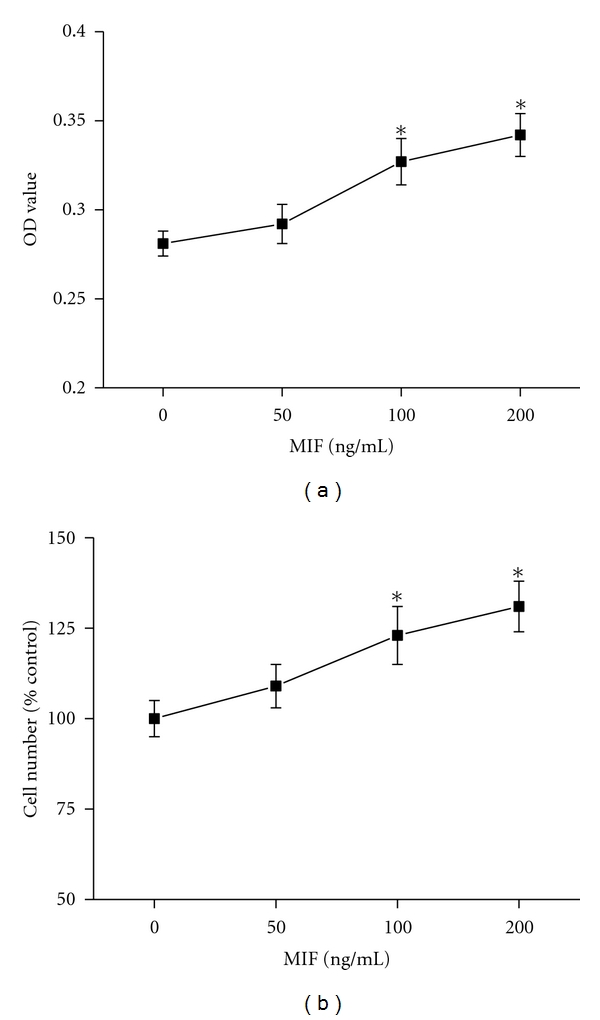
Effects of MIF on proliferation of rat PASMCs. Cell proliferation was demonstrated by MTT (a) and direct cell counting (b). MIF (100, 200 ng/mL) could directly promote PASMCs proliferation. *n* = 8. *P < 0.05 versus control.

It is known that hypoxia exposure significantly increases the PASMCs proliferation, but we found that this proliferation was obviously inhibited by three various concentrations of MIF antagonist ISO-1 (Figure 3, *P* < 0.05). ISO-1 had no effect on normoxic control PASMCs proliferation. Since we have established that hypoxia enhanced MIF expression and MIF contributed to the hypoxia-induced proliferation of PASMCs, we further tested the direct effect of MIF on PASMCs proliferation. Both MTT assay and cell counting showed that higher concentration (100, 200 ng/mL) of MIF could directly stimulate PASMCs proliferation (Figure 4).

### 3.4. MIF Stimulates PASMCs Proliferation through Activation of ERK1/2 and JNK

**Figure 5:**
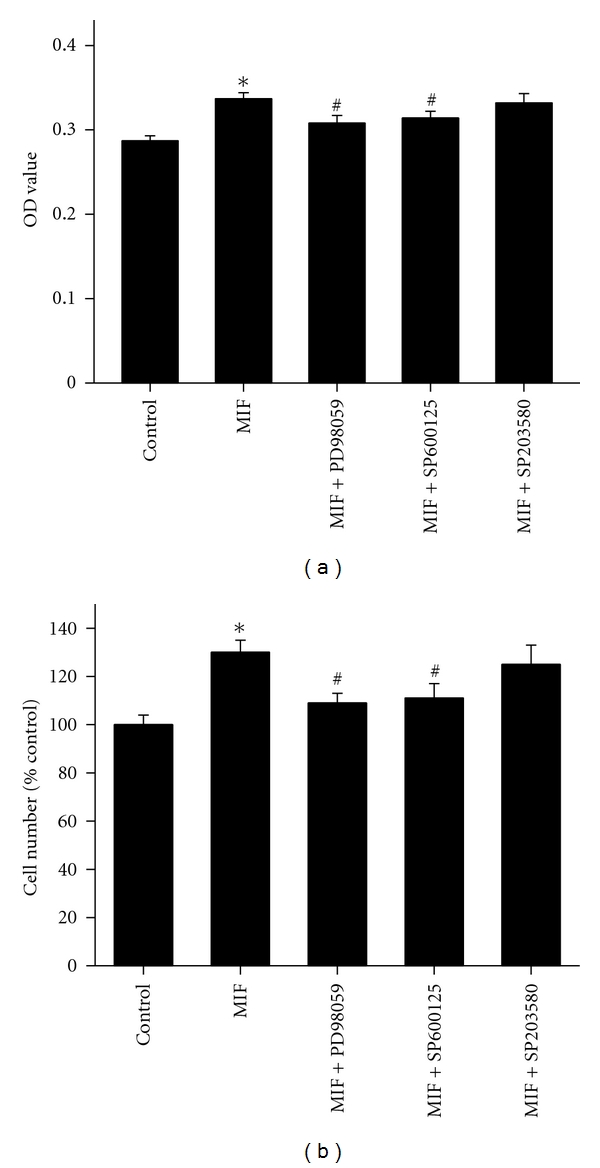
MIF-induced rat PASMCs proliferation is dependent on ERK1/2 and JNK activation. Cell proliferation was demonstrated by MTT (a) and direct cell counting (b). PASMCs proliferation stimulated by MIF (100 ng/mL) was blocked by the MEK inhibitor (PD 98059, 20 *μ*M) and JNK inhibitor (SP 600125, 20 *μ*M), whereas the p38 MAPK inhibitor (SB 203580, 20 *μ*M) had no effect. *n* = 8. *P < 0.05 versus vehicle-treated control; ^#^ *P* < 0.05 versus groups treated with MIF alone.

As shown in Figure 5(a), 100 ng/mL MIF-induced PASMCs proliferation was significantly blocked by specific MEK inhibitor PD 98059 and JNK inhibitor SP 600125 (*P* < 0.05), but not by p38 MAPK inhibitor SB 203580. The similar results were found as evaluated by cell counting (Figure 5(b)). These results suggested that ERK1/2 and JNK were involved in MIF proliferative pathway.

### 3.5. ERK1/2 and JNK Activation in MIF-Stimulated PASMCs

**Figure 6:**
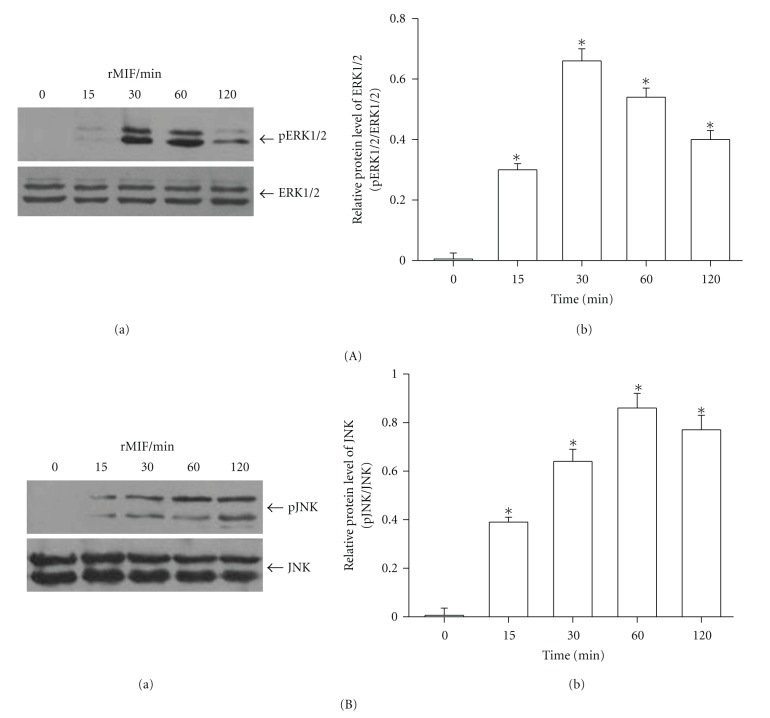
MIF induced ERK1/2 and JNK phosphorylation in rat PASMCs. PASMCs were treated with MIF (100 ng/mL) for different time points. Significant ERK1/2 (A) and JNK (B) phosphorylation was induced after 15 min of MIF treatment. (A) Cell lysates were subjected to immunoblotting analysis using antibodies against phospho-ERK1/2 (p ERK1/2) and total ERK1/2 (a). Bar graph showed ERK1/2 protein levels obtained from quantitative densitometry analysis (b). (B) Cell lysates were subjected to immunoblotting analysis using antibodies against phospho-JNK (p JNK) and total JNK (a). Bar graph showed JNK protein levels obtained from quantitative densitometry analysis (b). *n* = 5. *P < 0.05 versus control.

**Figure 7:**
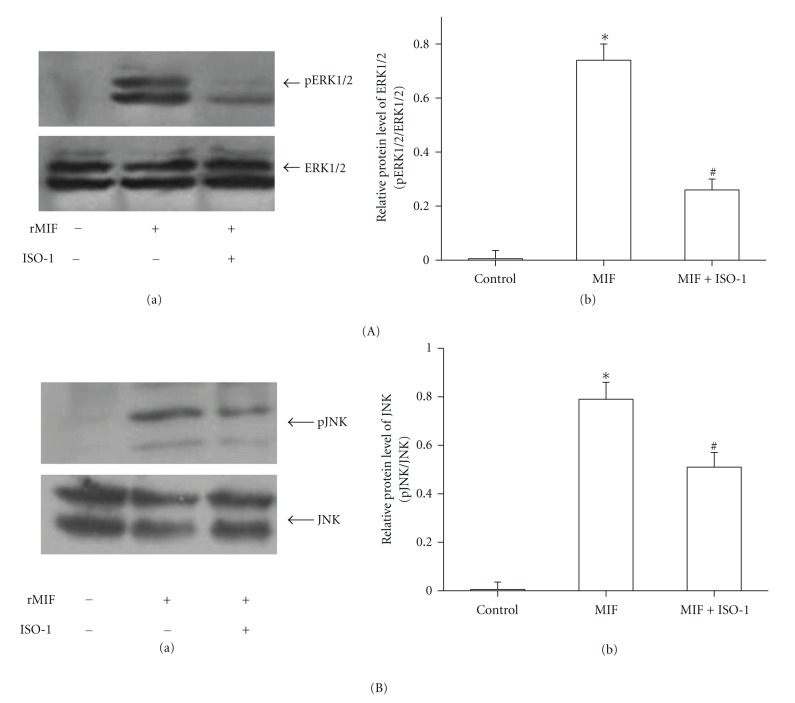
Effects of MIF inhibition on phosphorylation of ERK1/2 and JNK in rat PASMCs. MIF-stimulated ERK1/2 and JNK phosphorylation for 60 min was inhibited by MIF antagonist ISO-1 (50 *μ*M). (A) Cell lysates were analyzed by immunoblotting using antibodies specific for phospho-ERK1/2 (p ERK1/2) and total ERK1/2 (a). Bar graph showed ERK1/2 protein levels obtained from quantitative densitometry analysis (b). (B) Cell lysates were analyzed by immunoblotting using antibodies specific for phospho-JNK (p JNK) and total JNK (a). Bar graph showed JNK protein levels obtained from quantitative densitometry analysis (b). *n* = 6. *P < 0.05 versus vehicle-treated control; ^#^ *P* < 0.05 versus groups treated with MIF alone.

We found that MIF could activate phosphorylation of ERK1/2 and JNK. 100 ng/mL MIF-induced ERK1/2 activation was increased in a rapid time-dependent manner with maximal at 30 min and followed by a downregulation of ERK1/2 phosphorylation after 60 min (Figure 6(A)). MIF could also activate phosphorylation of JNK with peaks at 60 min (Figure 6(B)). Treatment with ISO-1 (50 *μ*M), MIF-induced both ERK1/2 and JNK activation was attenuated markedly (Figure 7).

## 4. Discussion

Chronic hypoxic exposure induces changes in the structure of pulmonary artery which is associated with increased pulmonary vascular resistance, pulmonary hypertension, and right heart failure. However, the mechanisms still remain unclear. The present study provides evidence that MIF is upregulated in the lungs of HPH rats and stimulates rats PASMCs proliferation, indicating a possible role for this cytokine in the pathogenesis of PH.

Investigations revealed that chronic hypoxia induced upregulation of gene expression of a wide spectrum of proinflammatory mediators, including chemokines and their receptors, cytokines, growth and differentiation factors, and adhesion and fibrosis-associated molecules [21]. It is increasingly appreciated that inflammatory mediators could directly contribute to the pulmonary vascular remodeling. They have significant effects on the local vascular wall cells, including increases in proliferation and matrix protein production [22]. It is reported that IL-6 may affect pulmonary vascular remodeling via direct stimulation of vascular smooth muscle cell (SMC) migration or by indirect effects on vascular SMC proliferation [23]. In addition, increased serum level of IL-1*β* was observed in the serum of patients with severe primary pulmonary hypertension [24]. These findings suggest that inflammatory mediators are closely associated with pulmonary hypertensive process.

As a critical proinflammatory cytokine, MIF is constitutively expressed in a variety of immune and nonimmune cells and also effectively secreted from various phenotype cells into the circulation [25]. Upon secretion, MIF exhibits broad regulatory properties, including stimulation of the growth of a number of cell lines, except for a key mediator in a number of immune and inflammatory diseases. Yang detected that MIF was a potent human-endothelial-cell-growth-promoting agent [26]. Neutralizing MIF bioactivity in atherosclerosis-susceptible mice reduces vascular SMC proliferation and neointimal thickening [19]. Additionally, Fu et al. found that MIF mediated the hypoxia response of vascular SMC, including cell migration and proliferation [27]. In human, it is reported that patients with PH showed higher MIF levels than patients without these manifestations in systemic sclerosis. It was suggested that MIF might contribute to vascular complications [28]. However, no studies have yet addressed the role of MIF in PASMCs proliferation in the pulmonary circulation exposed to hypoxia. Therefore, it will be of interest to explore whether MIF affects PASMCs proliferation during exposure to chronic hypoxia and contributes to the pulmonary vascular remodeling. In the current study, we found that both MIF mRNA and protein expression were increased in the lung tissues from HPH rats. Furthermore, the increased MIF mostly located in smooth muscle cells of large pulmonary arteries and endothelial cells of smaller PA, as well as the inflammatory cells and bronchial epithelial cells. These results indicated that MIF overexpression under hypoxia exposure condition might influence the cells in the vascular walls. Due to the key role of PASMCs in HPH, we examined the effect of MIF on hypoxia-induced PASMCs proliferation. As the results show, the isolated PASMCs proliferated in response to hypoxia, which was consistent with previous reports [29, 30]. Yet hypoxia-induced PASMCs proliferation was significantly attenuated by MIF antagonist ISO-1. ISO-1 is a small molecule inhibitor targeting MIF which inhibits the catalytic site of MIF and leads to a marked reduction in the biological function of MIF [31, 32]. Next, to observe the direct stimulatory effect of MIF on PASMCs growth, recombinant MIF was administrated to cells. We also found that MIF could promote PASMCs proliferation. However, there is evidence showing that proliferation of PASMCs was not affected by exogenous MIF [33]. The different isolated PASMCs may account for the difference in response to MIF. We also added 1% FBS to the culture medium which may contribute to the stimulated effect of MIF. Thus, our findings add MIF to the list of proliferative agents which may contribute to PASMCs proliferation in PH. Although MIF is a weak stimulator of proliferation, it might work in combination with something else such as other inflammatory mediators, or its receptor expression is altered during hypoxia exposure, which needs to be further studied.

Mitogen-activated protein kinases (MAPKs), including extracellular signal-regulated kinase (ERK), c-Jun NH2-terminal kinase (JNK), and p38 MAP kinase (p38), play the critical role in cell proliferation, survival, or apoptosis [34, 35]. Studies have implied that MIF-mediated signaling is associated with a sustained phosphorylation and activation of the p44/p42 ERK1/2 subfamily of MAPK [36, 37]. On the other hand, MIF could mediate phosphorylation of JNK in septic shock [38] and utilizes the JNK pathway in T cells and fibroblasts [39]. Also, MIF treatment strongly activated ERK1/2 and p38 MAPK in endometriotic cells [40]. Cheng et al. showed that MIF mediated adhesion molecule expression via the promotion of p38 MAPK activation in human endothelial cells [41]. Although MIF has been reported to activate MAPK signaling in several phenotype cells, there is no available information on the role of MAPK pathway in MIF-induced PASMCs response. Here, we demonstrated that MEK and JNK inhibitors block MIF-stimulated PASMCs growth, respectively. ISO-1 also inhibited MIF-activated phosphorylation of ERK1/2 and JNK. Thus, our findings support the idea that ERK1/2 and JNK signaling participate in the MIF proliferative pathway. But our findings also show that p38 MAPK did not appear to contribute to MIF-promoted PASMCs proliferation. The previous findings of Amin reported that MIF-induced migration of human dermal microvascular endothelial cells was not blocked by inhibitors of Src and p38 MAPK [42]. It indicated that MIF mediated the various effects through different signal transduction pathways in different cell types.

In summary, the current study showed that MIF expression was increased in the lungs from HPH rats. This cytokine may act as a growth factor for PASMCs partially through ERK1/2 and JNK pathway without the involvement of p38 MAPK. We conclude that MIF contributes to the hypoxic pulmonary hypertension, but further studies will be needed to extend these findings, with the aim of identifying new therapeutic targets for treatment of pulmonary hypertension.

## Figures and Tables

**Table 1:** Haemodynamic variables and right ventricle hypertrophy index.

Group	*n*	RVSP (mmHg)	RV/(LV+S)
Control	8	23 ± 0.5	0.26 ± 0.02
Hypoxia	10	48 ± 2.8*	0.41 ± 0.05*

Rats were housed intermittently in a hypobaric hypoxia chamber containing 10% oxygen and exposed to these conditions for 10 h/d continuing 28 days. Right ventricle systolic pressure (RVSP) and the ratios of right ventricle/[left ventricle + septum] (RV/[LV+S]) weight were examined as indicators of pulmonary hypertension. Values are means ± SD. *P < 0.05, compared with the corresponding value in control rats.
